# Voltage-gated sodium channels in taste bud cells

**DOI:** 10.1186/1471-2202-10-20

**Published:** 2009-03-12

**Authors:** Na Gao, Min Lu, Fernando Echeverri, Bianca Laita, Dalia Kalabat, Mark E Williams, Peter Hevezi, Albert Zlotnik, Bryan D Moyer

**Affiliations:** 1 Senomyx, Inc, 4767 Nexus Centre Drive, San Diego, CA 92121, USA; 2 Current address: Department of Physiology and Biophysics, University of California Irvine, Irvine, California 92697, USA

## Abstract

**Background:**

Taste bud cells transmit information regarding the contents of food from taste receptors embedded in apical microvilli to gustatory nerve fibers innervating basolateral membranes. In particular, taste cells depolarize, activate voltage-gated sodium channels, and fire action potentials in response to tastants. Initial cell depolarization is attributable to sodium influx through TRPM5 in sweet, bitter, and umami cells and an undetermined cation influx through an ion channel in sour cells expressing PKD2L1, a candidate sour taste receptor. The molecular identity of the voltage-gated sodium channels that sense depolarizing signals and subsequently initiate action potentials coding taste information to gustatory nerve fibers is unknown.

**Results:**

We describe the molecular and histological expression profiles of cation channels involved in electrical signal transmission from apical to basolateral membrane domains. TRPM5 was positioned immediately beneath tight junctions to receive calcium signals originating from sweet, bitter, and umami receptor activation, while PKD2L1 was positioned at the taste pore. Using mouse taste bud and lingual epithelial cells collected by laser capture microdissection, SCN2A, SCN3A, and SCN9A voltage-gated sodium channel transcripts were expressed in taste tissue. SCN2A, SCN3A, and SCN9A were expressed beneath tight junctions in subsets of taste cells. SCN3A and SCN9A were expressed in TRPM5 cells, while SCN2A was expressed in TRPM5 and PKD2L1 cells. HCN4, a gene previously implicated in sour taste, was expressed in PKD2L1 cells and localized to cell processes beneath the taste pore.

**Conclusion:**

SCN2A, SCN3A and SCN9A voltage-gated sodium channels are positioned to sense initial depolarizing signals stemming from taste receptor activation and initiate taste cell action potentials. SCN2A, SCN3A and SCN9A gene products likely account for the tetrodotoxin-sensitive sodium currents in taste receptor cells.

## Background

Taste buds house specialized neuroepithelial cells that sense and transmit information regarding the composition of food [1]. These taste cells express receptors for sweet, bitter, umami (the savory taste of glutamate), sour and salty tastants in apical microvilli facing the saliva [2,3]. Following taste receptor activation, taste cells depolarize and fire action potentials resulting in the release of neurotransmitters to nerve fibers innervating basolateral membranes [4,5]. Segregation of taste receptors at the taste pore from the machinery involved in transmitting signals to nerve fibers is mediated by tight junctions, specialized protein networks that separate apical and basolateral membrane domains [6].

Information regarding the composition of food flows from the apical to the basolateral domain in taste receptor cells. Binding of tastants to apical sweet, bitter, and umami G protein-coupled receptors activates a signal transduction pathway involving the heterotrimeric G protein alpha subunit gustducin, phospholipase C β2, the type 3 inositol 1, 4, 5-triphosphate receptor and calcium release from intracellular stores [5]. Calcium, in turn, activates the monovalent-selective cation channel TRPM5, and the resultant sodium influx depolarizes sweet, bitter, and umami taste cells [7-9]. In contrast, sour tastants likely directly gate an acid-sensitive ion channel leading to cation influx and sour taste cell depolarization; PKD2L1 is a candidate sour taste receptor [10,11]. Salty taste encompasses amiloride-sensitive and amiloride-insensitive components. The amiloride-sensitive pathway may require an epithelial sodium channel, however the precise target of amiloride is undefined, and the amiloride-insensitive pathway may require a non-selective cation channel expressed in nerve elements [12-16]. Specialized voltage sensors, the voltage-gated sodium channels, detect membrane depolarization and initiate the rising phase of action potentials that code tastant information to afferent gustatory nerve fibers [17]. Despite the observance of voltage-gated sodium currents in taste receptor cells for the past two decades [18-24], the molecular identity of the voltage-gated sodium channel gene products that sense depolarizing stimuli and initiate taste cell action potentials are unknown.

We describe the molecular and histological expression profiles of cation channels involved in transmitting information from apical taste receptors to basolateral nerve fibers. SCN2A, SCN3A and SCN9A voltage-gated sodium channels were identified in sweet, bitter, umami, and sour taste cell populations. SCN2A, SCN3A and SCN9A channels are, therefore, positioned to sense depolarizing stimuli stemming from taste receptor activation and initiate action potentials coding tastant information.

## Results

### Localization of TRPM5 and PKD2L1 in taste receptor cells

**Figure 1:**
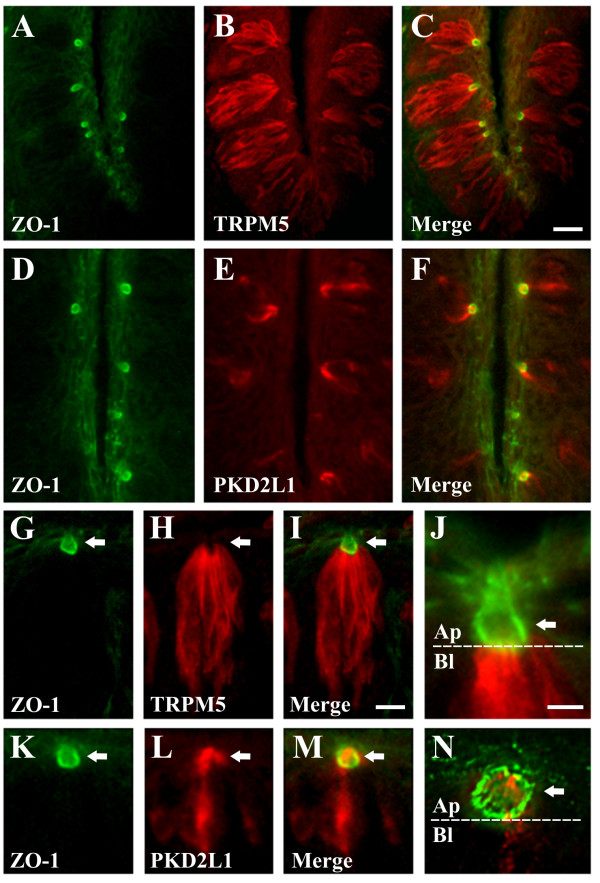
**Localization of TRPM5 and PKD2L1 in taste receptor cells**. A-C and G-I, TRPM5 immunoreactivity is concentrated in taste receptor cell processes below the taste pore. Double label IHC for the tight junction protein ZO-1 (A, G) and TRPM5 (B, H) reveals that TRPM5 immunoreactivity is localized to basolateral membranes beneath tight junctions in the merged images (C, I). D-F and K-M, PKD2L1 immunoreactivity is concentrated in taste receptor cell processes at the taste pore. Double label IHC for the tight junction protein ZO-1 (D, K) and PKD2L1 (E, L) reveals that PKD2L1 immunoreactivity is localized, in part, to apical membranes above tight junctions in the merged images (F, M). Images are from CV papilla and are longitudinal sections. Images in A-F illustrate low magnification of multiple taste buds; images in G-I and K-M illustrate high magnification of single taste buds with the taste pore at the top. J, Zoom of taste pore region. TRPM5 immunoreactivity (red) is prevalent in basolateral membranes (Bl) beneath tight junctions, labelled with ZO-1 (green), but absent from apical membranes (Ap) above tight junctions. N, Zoom of taste pore region. PKD2L1 immunoreactivity (red) is enriched in Ap membranes above tight junctions. Arrows denote taste pore region and apical side of taste bud. White dashed line illustrates boundary between apical and basolateral membrane domains. Scale bar is 20 μm in C and represents scale for A-F, 10 μm in I and represents scale for G-I and K-M, and 5 μm in J and represents scale for J and N.

We localized TRPM5, a calcium-activated monovalent-selective cation channel that opens following tastant activation of sweet, bitter, and umami G protein-coupled receptors [7-9], and PKD2L1, an ion channel in sour taste cells [10,11,25], in relation to ZO-1, a component of tight junctions that separate apical and basolateral plasma membrane domains [6,26]. TRPM5 immunoreactivity was polarized beneath tight junctions to the basolateral membrane region and present in cell bodies of taste bud cells (Fig. 1A–C), whereas PKD2L1 immunoreactivity was enriched in taste cell processes that extend to the apical membrane and present in cell bodies of taste bud cells (Fig. 1D–F). Careful examination revealed that TRPM5 immunolabelling was abundant in lateral membrane domains immediately beneath tight junctions but absent from the apical taste pore (Fig. 1G–J). PKD2L1 immunolabelling, by contrast, was present at the apical taste pore region above tight junctions (Fig. 1K–N).

### Taste bud cells express SCN2A, SCN3A and SCN9A voltage-gated sodium channel genes

**Figure 2:**
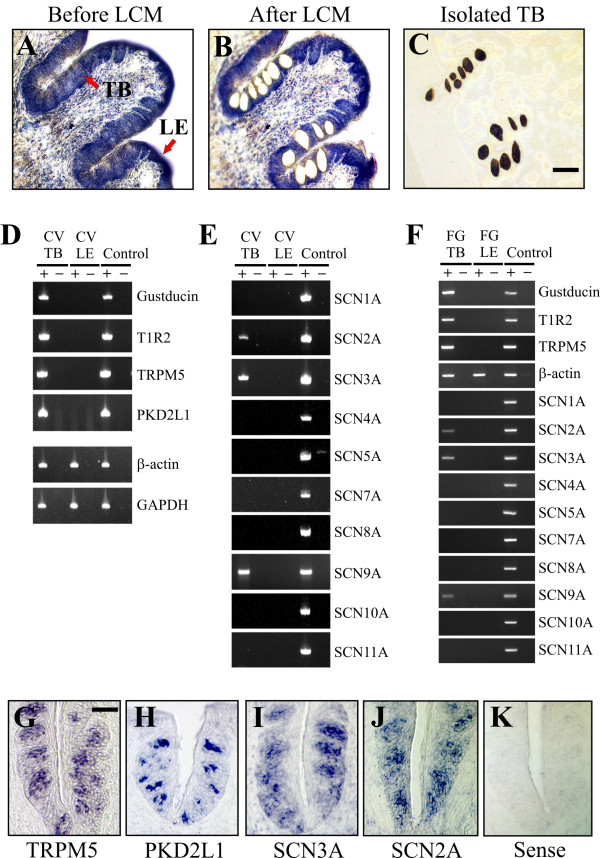
**SCN2A, SCN3A and SCN9A are taste bud-associated genes**. A-C, Isolation of mouse taste cells by LCM. CV papilla containing multiple taste bud areas before LCM (A), the same region after LCM (B), and the isolated taste bud areas (C). TB denotes taste bud and LE denotes lingual epithelium. LE adjacent to CV papilla and devoid of taste buds was also collected by LCM. Since tissue sections are not coverslipped and come into contact with an adhesive cap to remove collected regions during LCM, small lighting differences and focus changes are observed between images. Scale bar is 50 μm. D-F, RT-PCR amplification of transcripts from TB and LE collected by LCM. The taste bud-associated genes gustducin, T1R2, TRPM5, and PKD2L1 are expressed in TB but not LE, whereas the housekeeping genes β-actin and GAPDH are expressed in both TB and LE (D, F). SCN2A, SCN3A and SCN9A are taste bud-associated genes and are expressed in CV TB (E) and FG TB (F) but not LE. All primer sets amplified products from a pool of fifteen diverse tissues, denoted as 'Control' in the figure (Mouse Total RNA Master Panel; Clontech). Cloning and sequencing of PCR products confirmed that amplified bands corresponded to the expected genes. '+' indicates reverse transcription and '-' indicates no reverse transcription was performed. PCR products were only obtained following reverse transcription, indicating that bands originated from mRNA and not genomic DNA. G-K, ISH for TRPM5 (F), PKD2L1 (H), SCN3A (I), SCN2A (J) illustrating expression of transcripts in CV taste buds. Images in G-J depict antisense probes and image in K depicts a representative sense probe. No signals were observed with control sense probes. Scale bar in F is 50 μm and represents scale for G-K.

Taste bud cells depolarize, activate voltage-gated sodium channels, and fire action potentials in response to tastants [21,23,27-29]. Initial cell depolarization is attributed to sodium influx through TRPM5 in sweet, bitter, and umami taste cells and an undetermined cation influx through an ion channel in sour taste cells expressing PKD2L1 [9-11]. Microarray analyses revealed voltage-gated sodium, potassium, and calcium channel transcripts prevalent in mouse taste buds (data not shown). To elucidate the molecular identities of the voltage-gated sodium channel(s) activated by these depolarizing stimuli, mouse taste bud and lingual epithelial cells were collected by laser capture microdissection (LCM) (Fig. 2A–C) and analyzed by RT-PCR. Gustducin, a G protein involved in sweet, umami, and bitter taste signalling [30,31], T1R2, a component of the sweet taste receptor [32,33], TRPM5, and PKD2L1 were exclusively expressed in taste buds and absent from lingual epithelial cells, demonstrating efficient isolation of taste tissue by LCM (Fig. 2D). The housekeeping genes β-actin and GAPDH were expressed in taste buds and lingual epithelial cells validating that intact RNA was isolated from both regions (Fig. 2D).

The voltage-gated sodium channel family is comprised of ten members: SCN1A (Nav1.1), SCN2A (Nav1.2; SCN2A1 in mouse), SCN3A (Nav1.3), SCN4A (Nav1.4), SCN8A (Nav1.6), and SCN9A (Nav1.7) are blocked by tetrodotoxin, whereas SCN5A (Nav1.5), SCN7A (Nax), SCN10A (Nav1.8), and SCN11A (Nav1.9) are not blocked by tetrodotoxin [17,34,35]. SCN2A, SCN3A and SCN9A were specifically expressed in circumvallate (CV) taste buds in the back of the tongue (Fig. 2E) and fungiform (FG) taste buds in the front of the tongue (Fig. 2F) but not detectable in lingual epithelial cells by RT-PCR analysis. SCN3A was the thirteenth top expressed taste bud-associated gene in microarray studies. Other SCN genes were not readily detectable in taste buds (Fig. 2E, F). Although primer sets covered known splice variants described in Genbank, it is conceivable that novel splice variants may increase the functional repertoire of voltage-gated sodium channels in taste buds. Taste bud expression of SCN3A and SCN2A transcripts was validated by in situ hybridization (ISH). SCN3A and SCN2A antisense probes labelled taste buds cells, similar to TRPM5 and PKD2L1 probes, while control sense probes yielded no signal (Fig. 2G–K). SCN9A expression was not detected by ISH (data not shown). Thus, SCN2A, SCN3A and SCN9A are taste bud-associated genes.

**Figure 3:**
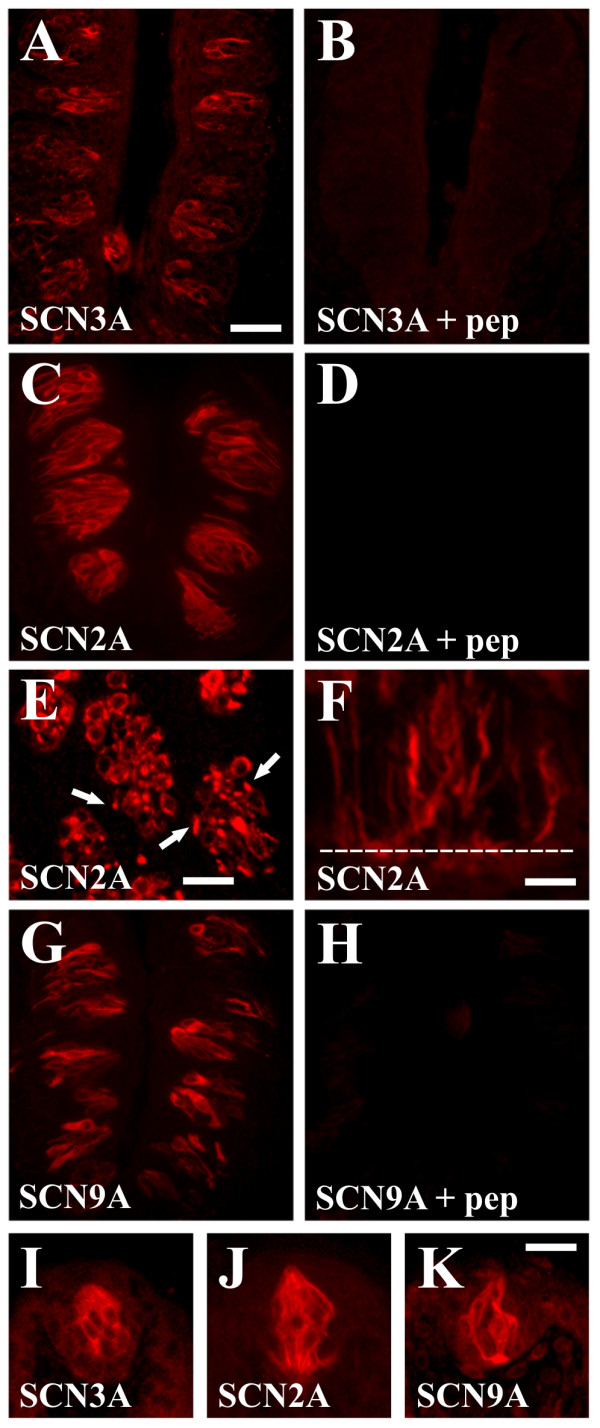
**Expression of SCN2A, SCN3A, and SCN9A in taste buds by IHC**. A, SCN3A is expressed in a population of CV taste cells. B, SCN3A peptide blocks taste cell labelling. C, SCN2A is expressed in a population of CV taste cells. D, SCN2A peptide blocks taste cell labelling. E-F, SCN2A is also expressed in nerve fibers innervating taste cells. E, Transverse section with SCN2A immunoreactivity prevalent in punctuate nerve fibers (arrows) as well as taste cells. F, Longitudinal section at base of taste bud with SCN2A immunoreactivity in nerve fibers near the basal lamina (dotted white line). G, SCN9A is expressed in a population of CV taste cells. H, SCN9A peptide blocks taste cell labelling. I-K, SCN3A (I), SCN2A (J), and SCN9A (K) are expressed in FG taste cells. Scale bar is 40 μm in A and represents scale for A-D and G-H, 20 μm in E, 5 μm in F, and 25 μm in I-K.

Immunohistochemistry (IHC) studies were performed to evaluate SCN2A, SCN3A and SCN9A protein expression in taste buds. Antisera to SCN3A and SCN9A labelled a subset of taste cells (5.3 +/- 0.5 cells/section for SCN3A; 6.0 +/- 0.4 cells/section for SCN9A), whereas antisera to SCN2A labelled numerous taste cells in CV taste buds (11.0 +/- 0.5 cells/section) (Fig 3A, C, G). Signals were localized to cell bodies and processes below tight junctions; labelling was not observed at the apical taste pore. Preincubation of antisera with the relevant immunizing peptide abolished taste cell labelling (Fig. 3B, D, H). In addition to taste cells, SCN2A antisera also labelled nerve fibers innervating taste cells (Fig. 3E) and the nerve plexus below taste buds and near the basal lamina (Fig. 3F), similar to the reported neuronal markers syntaxin-1 and synaptobrevin-2 [36,37]. SCN2A, SCN3A, and SCN9A antisera also labelled FG taste bud cells (Fig. 3I–K).

### SCN2A, SCN3A and SCN9A are expressed in specific taste cell populations

**Figure 4:**
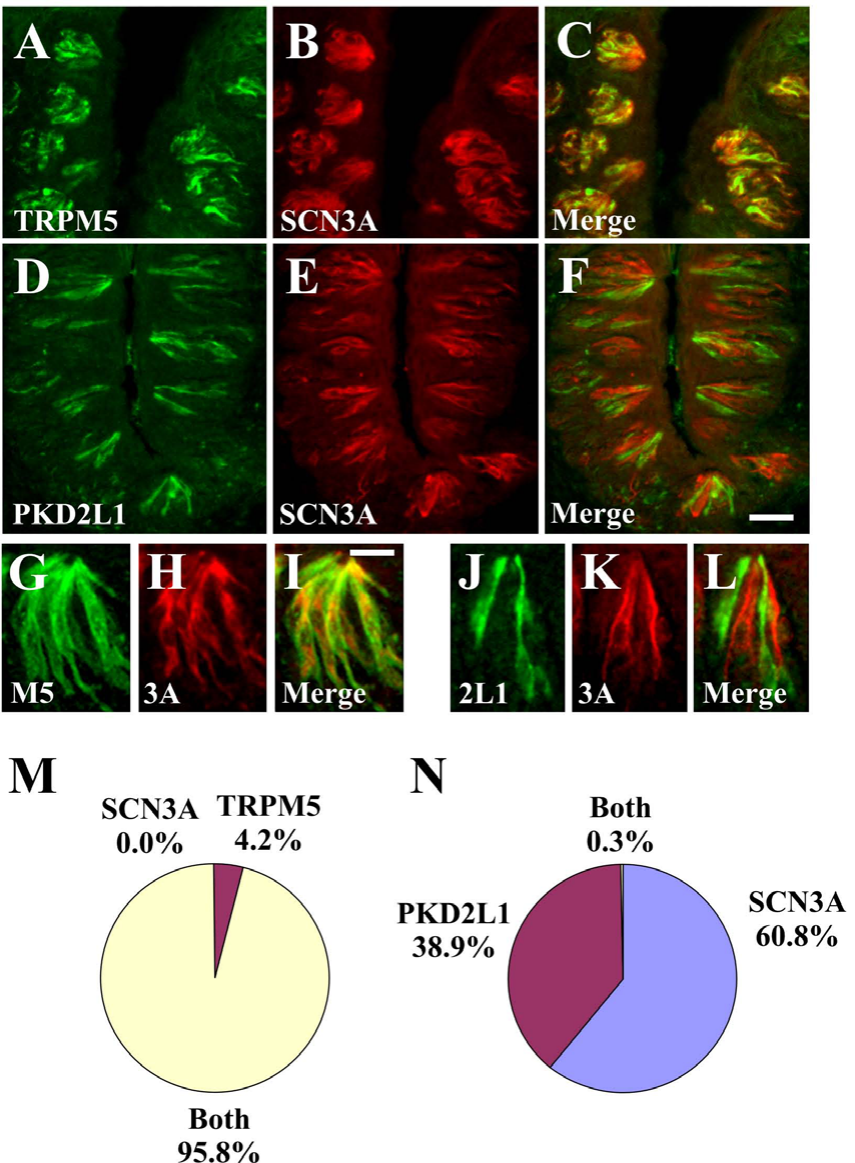
**SCN3A is expressed in TRPM5 but not PKD2L1 cells**. A-C and G-I, Double label IHC for TRPM5 (A, G) and SCN3A (B, H) demonstrating SCN3A immunoreactivity in TRPM5 cells. TRPM5 and SCN3A signals label the same cells in the merged images (C, I). D-F and J-L, Double label IHC for PKD2L1 (D, J) and SCN3A (E, K) demonstrating SCN3A immunoreactivity is absent from PKD2L1 cells. PKD2L1 and SCN3A signals label distinct cells in the merged images (F, L). Quantitation of SCN3A expression in TRPM5 cells (M) and PKD2L1 cells (N). Images are from CV papilla and are longitudinal sections. Images in A-F illustrate multiple taste buds; images in G-L illustrate single taste buds with the taste pore at the top. Scale bar is 40 μm in F and represents scale for A-F, and 15 μm in I and represents scale for G-L.

**Figure 5:**
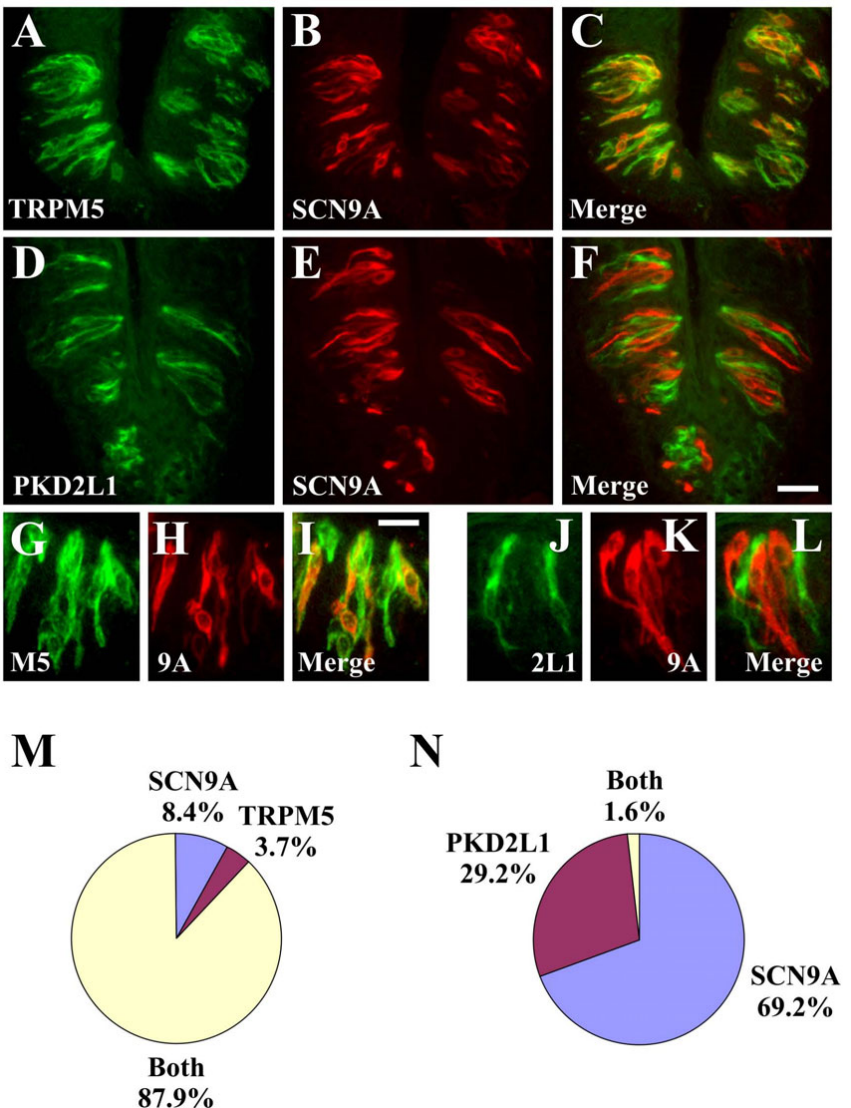
**SCN9A is expressed in TRPM5 but not PKD2L1 cells**. A-C and G-I, Double label IHC for TRPM5 (A, G) and SCN9A (B, H) demonstrating SCN9A immunoreactivity in TRPM5 cells. TRPM5 and SCN9A signals label similar cells in the merged images (C, I). D-F and J-L, Double label IHC for PKD2L1 (D, J) and SCN9A (E, K) demonstrating SCN9A immunoreactivity is absent from PKD2L1 cells. PKD2L1 and SCN9A signals label distinct cells in the merged images (F, L). Quantitation of SCN9A expression in TRPM5 cells (M) and PKD2L1 cells (N). Images are from CV papilla and are longitudinal sections. Images in A-F illustrate multiple taste buds; images in G-L illustrate single taste buds with the taste pore at the top. Scale bar is 30 μm in F and represents scale for A-F, and 15 μm in I and represents scale for G-L.

**Figure 6:**
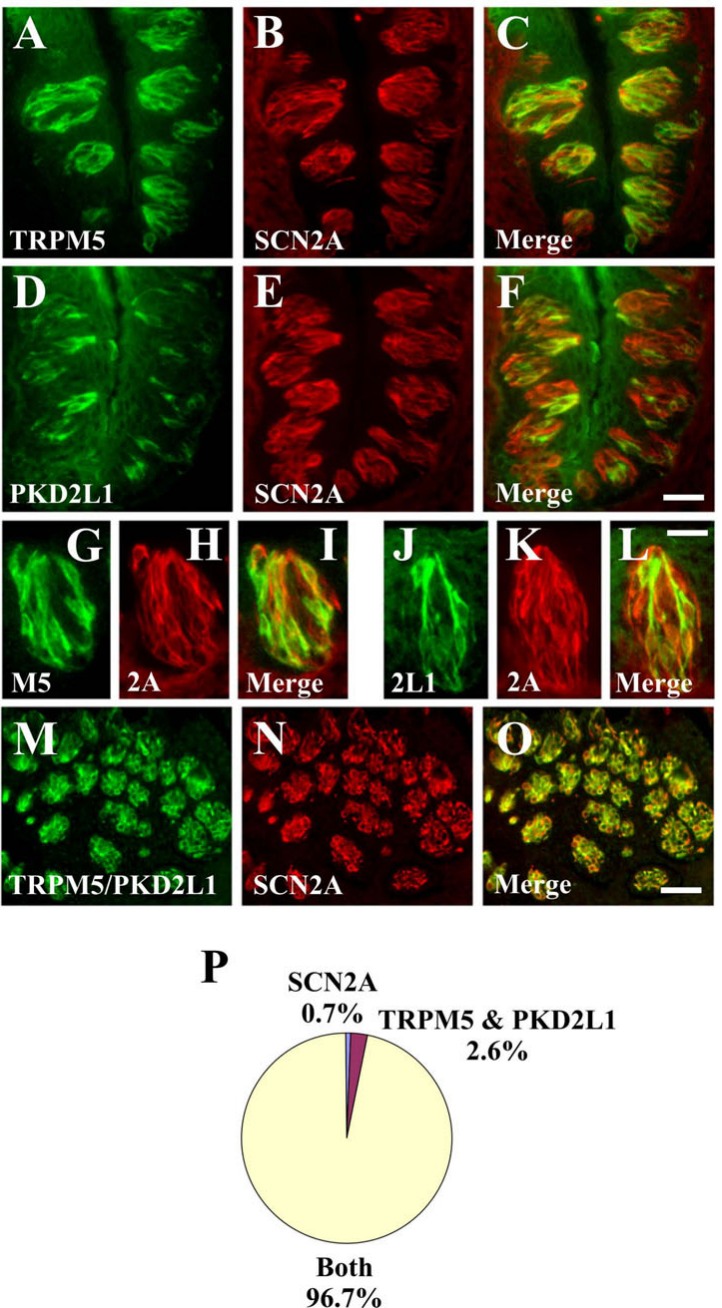
**SCN2A is expressed in TRPM5 and PKD2L1 cells**. A-C and G-I, Double label IHC for TRPM5 (A, G) and SCN2A (B, H) demonstrating SCN2A immunoreactivity in TRPM5 cells. TRPM5 signals are associated with SCN2A signals; however, many SCN2A cells do not express TRPM5 in the merged images (C, I). D-F and J-L, Double label ISH for PKD2L1 (D, J) and SCN2A (E, K) demonstrating SCN2A immunoreactivity in PKD2L1 cells. PKD2L1 signals are associated with SCN2A signals; however, many SCN2A cells do not express PKD2L1 in the merged images (F, L). M-N, Double label IHC for TRPM5 with PKD2L1 (M) and SCN2A (N) demonstrating SCN2A immunoreactivity in TRPM5 and PKD2L1 cells. Combined TRPM5 and PKD2L1 signals are associated with SCN2A signals in the merged image (O). Quantitation of SCN2A expression in TRPM5 and PKD2L1 cells (P). Images in A-F and M-O illustrate multiple taste buds; images in G-L illustrate single taste buds with the taste pore at the top. Images are from CV papilla and are longitudinal sections (A-L) or transverse sections (M-O). Scale bar is 40 μm in F and represents scale for A-F, 20 μm in L and represents scale for G-L, and 50 μm in O and represents scale for M-O.

Voltage-gated sodium channel currents have been recorded in TRPM5 cells (sweet, bitter, and umami; type II cells) as well as PKD2L1 cells (sour; type III cells) [18-20,38,39]. Therefore, double label IHC studies were conducted to determine the specific taste cell population(s) expressing SCN2A, SCN3A and SCN9A. To this end, we first validated that TRPM5 and PKD2L1 antisera labelled distinct cell types (2.3% TRPM5 and PKD2L1; 61.4% TRPM5 only; 36.4% PKD2L1 only) as previously reported using ISH analyses [10,11]. There were 6.4 +/- 0.2 TRPM5 cells and 3.7 +/- 0.2 PKD2L1 cells per section. SCN3A immunoreactivity was present in TRPM5 cells (95.8% SCN3A and TRPM5; 4.2% TRPM5 only; 0.0% SCN3A only) indicating that SCN3A is a voltage gated sodium channel in sweet, bitter, and umami cells (Fig. 4A–C, G–I, M). By contrast, SCN3A immunoreactivity was absent from PKD2L1 cells (0.3% SCN3A and PKD2L1; 38.9% PKD2L1 only; 60.8% SCN3A only), demonstrating that SCN3A is not expressed in sour cells (Fig. 4D–F, J–L, N). Similar to SCN3A, SCN9A immunoreactivity was present in TRPM5 cells (87.9% SCN9A and TRPM5; 3.7% TRPM5 only; 8.4% SCN9A only) (Fig. 5A–C, G–I, M) and absent from PKD2L1 cells (1.6% SCN9A and PKD2L1; 29.2% PKD2L1 only; 69.2% SCN9A only) (Fig. 5D–F, J–L, N). SCN2A immunoreactivity was present in TRPM5 cells (Fig. 6A–C, G–I) as well as PKD2L1 cells (Fig. 6D–F, J–L). Due to the abundance of SCN2A labelling, it was difficult to quantitate expression in separate TRPM5 and PKD2L1 cell populations. When TRPM5 and PKD2L1 antisera were combined, nearly all cells coexpressed SCN2A (Fig. 6M–O, P) (96.7% TRPM5/PKD2L1 and SCN2A; 2.6% TRPM5/PKD2L1 only; 0.7% SCN2A only), indicating that SCN2A is a voltage-gated sodium channel in sweet, bitter, umami, and sour cells.

### HCN4 is expressed in PKD2L1 cells

**Figure 7:**
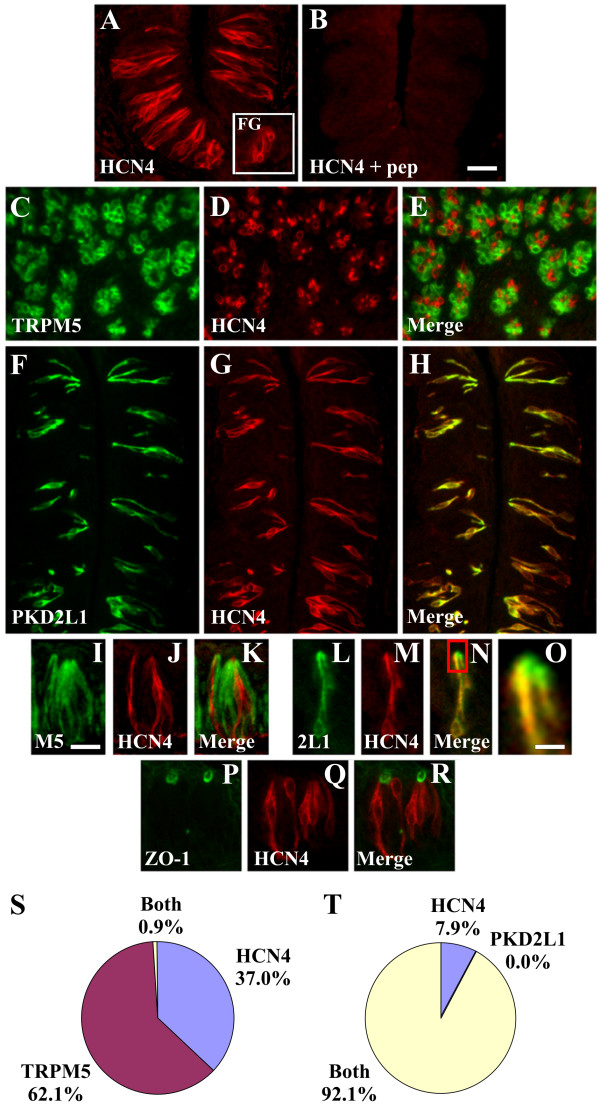
**HCN4 is expressed in PKD2L1 but not TRPM5 cells**. A, HCN4 is expressed in a population of CV taste cells by IHC. Inset shows expression of HCN4 in FG taste cells. B, HCN4 peptide blocks taste cell labelling. C-E and I-K, Double label IHC for TRPM5 (C, I) and HCN4 (D, J) demonstrating absence of HCN4 immunoreactivity in TRPM5 cells. TRPM5 and HCN4 signals label distinct cells in the merged images (E, K). F-H and L-N, Double label IHC for PKD2L1 (F, L) and HCN4 (G, M) demonstrating HCN4 immunoreactivity in PKD2L1 cells. PKD2L1 and HCN4 signals label the same cells in the merged images (H, N). O, Magnification of apical taste bud region from boxed area in N. PKD2L1 immunoreactivity extends to the apical taste pore whereas HCN4 immunoreactivity terminates beneath the PKD2L1 signal. P-R, Double label IHC for ZO-1 (P) and HCN4 (Q) demonstrating HCN4 localization below tight junctions in the merged image (R). Quantitation of HCN4 expression in TRPM5 cells (S) and PKD2L1 cells (T). Images are from CV papilla and are longitudinal sections except for C-E which are transverse sections. Images in A-H illustrate multiple taste buds; images in I-N and P-R illustrate one to two taste buds with the taste pore at the top. Scale bar is 25 μm in B and represents scale for A-H, 15 μm in I and represents scale for I-N and P-R, and 5 μm in O.

The hyperpolarization-activated and cyclic nucleotide-gated cation channels HCN1 and HCN4 were proposed as a candidate sour taste receptor [40]. Since cesium, a blocker of HCN channels, did not attenuate taste cell responses to sour stimuli, it is unlikely that HCN channels are the primary sensor for acidic tastants [41]. HCN channels could, however, modulate sour taste cell function. We first tested if HCN transcripts were expressed in taste buds by RT-PCR analysis as reported previously [40]. HCN4 transcripts were detected in taste buds isolated by LCM (data not shown). We next conducted IHC experiments to evaluate HCN4 expression in taste cell populations. HCN4 antisera labelled a subset of FG and CV taste cells (3.5 +/- 0.3 cells/section) and signals were blocked by preincubation with the immunizing peptide (Fig. 7A–B). Double labelling studies revealed that HCN4 immunoreactivity was absent from TRPM5 cells (Fig. 7C–E, I–K, S) and present in PKD2L1 cells (Fig. 7F–H, L–N, T). Cells expressing HCN4 did not express TRPM5 (0.9% HCN4 and TRPM5; 62.1% TRPM5 only; 37.0% HCN4 only) but coexpressed PKD2L1 (92.1% HCN4 and PKD2L1; 0.0% PKD2L1 only; 7.9% HCN4 only), indicating that HCN4 is expressed in the sour taste cell population. Careful examination revealed that HCN4 immunoreactive taste cell processes terminated below the apical membrane, labelled with PKD2L1 (Fig. 7O), and below tight junctions, labelled with ZO-1 (Fig. 7P–R). Thus, analogous to TRPM5, HCN4 is expressed in membrane compartments beneath tight junctions.

## Discussion

Identification of the voltage-gated sodium channels in taste receptor cells has important implications for understanding taste cell signal transduction and the transmission of tastant information from apical taste receptors to basolateral nerve fibers. We identified transcripts for the tetrodotoxin-sensitive SCN2A, SCN3A, and SCN9A voltage-gated sodium channels in taste buds isolated by laser capture microdissection. Therefore, SCN2A, SCN3A, and SCN9A gene products likely generate the tetrodotoxin sensitive voltage-gated sodium channel currents recorded from taste cells [21-24]. Notably, transcripts for the tetrodotoxin-resistant voltage-gated sodium channels SCN5A, SCN7A, SCN10A and SCN11A were not detected in taste buds, in line with the absence of tetrodotoxin-resistant voltage-gated sodium currents in taste cells [19,22]. SCN3A and SCN9A were specifically expressed in TRPM5 (sweet, bitter, and umami) cells whereas SCN2A was expressed in TRPM5 and PKD2L1 (sour) cells. We propose that SCN2A, SCN3A, and SCN9A sense the initial depolarizing stimuli stemming from taste receptor activation at the apical membrane and initiate action potentials coding tastant information to gustatory nerve fibers at the basolateral membrane.

In response to tastants, taste bud cells depolarize, activate voltage-gated sodium channels, and fire action potentials [21,23,27-29]. An initial cell depolarization, referred to as a receptor potential, is attributed to sodium influx through TRPM5 in sweet, bitter, and umami cells [9]. Therefore, precise spatial localization of the calcium-activated TRPM5 cation channel is essential for efficient detection of calcium signals stemming from sweet, bitter, and umami taste receptor activation. We localized TRPM5 to the basolateral membrane domain and, more specifically, observed polarization of TRPM5 immediately beneath tight junctions. Enrichment of TRPM5 in lateral cell processes below the taste pore would simultaneously allow for efficient detection of calcium-signals associated with apical taste receptor stimulation and permit sodium influx from the sodium-replete interstitial fluid bathing taste cell basolateral membranes. Importantly, TRPM5 immunoreactivity was absent from apical microvilli, consistent with polarization of TRPM5 to the basolateral membrane in chemosensory cells of the respiratory system and gastrointestinal tract [42]. Opening apical membrane TRPM5 channels would not efficiently depolarize taste cells due to the lack of a strong electrochemical driving force for sodium entry from saliva, which exhibits a naturally low sodium concentration [43].

In contrast to sweet, bitter, and umami taste cells, where taste receptor activation initiates a signal transduction cascade leading to cell depolarization [5], sour taste cell depolarization is likely mediated by direct gating of an ion channel by acids in cells expressing PKD2L1 [10,11]. We localized PKD2L1 to the apical taste pore region above tight junctions. Expression of PKD2L1 at the apical membrane would allow sour cells to directly sample saliva for acids. Since PKD2L1 generates a non-selective cation channel permeable to monovalent and divalent ions, [11,44], influx of protons, from acidic tastants, or calcium, from saliva, down their respective electrochemical gradients would directly depolarize sour cells. Application of acids to the taste pore results in an influx of extracellular calcium in sour cells, suggesting that calcium may constitute a depolarizing signal [41,45].

HCN channel transcripts and acid-sensitive hyperpolarization-activated currents have been identified in taste cells, suggesting that HCN channels may constitute a sour taste receptor [20,40]. This hypothesis is not supported by findings that cesium, an inhibitor of HCN currents, did not affect taste cell responses to sour stimuli [41]. We specifically localized HCN4 to PKD2L1 (sour) taste cells. Polarization of HCN4 to cell processes below the apical membrane indicates that HCN4 is not a primary sour detector; however, HCN4 may modulate depolarizing signals originating at the apical membrane. As HCN4 inward currents are augmented by low pH [40], interstitial sodium influx through HCN4 may bolster depolarizing stimuli and generate pacemaker currents important for action potential generation in sour cells [46]. Alternatively, since HCN4 currents are modulated by cAMP [47], activators of stimulatory and inhibitory G-proteins could affect HCN4 inward currents and regulate sour taste cell activation [48,49].

We identified multiple voltage-gated sodium channel transcripts in isolated taste buds. IHC studies revealed that SCN3A and SCN9A were expressed in TRPM5 (sweet, bitter, and umami) cells while SCN2A was expressed in TRPM5 and PKD2L1 (sour) cells. Sodium channels localized to cell bodies and basolateral membrane domains, where they are positioned to sense the initial depolarization from taste receptors. Influx of sodium from the interstitial fluid through SCN2A, SCN3A, and SCN9A would lead to the rising phase of action potentials. Since the taste cell resting membrane potential is close to the threshold voltage for action potential generation [24], a small initial depolarization from TRPM5 and the sour taste receptor would result in a large subsequent depolarization by voltage-gated sodium channels. TRPM5 cells express three tetrodotoxin-sensitive sodium channels, SCN2A, SCN3A, and SCN9A. Neurons and neuroepithelial cells frequently express multiple voltage-gated sodium channels [50-52]. Sequestration of channels to specialized microdomains in neurons allows compartmentalization of channel function. SCN2A and SCN8A localize to axon initial segments and nodes of Ranvier for action potential initiation, whereas SCN1A and SCN3A localize to the somatodendritic region, for integration of post-synaptic potentials and action potential back-propagation [53]. In this manner, SCN2A, SCN3A, and SCN9A may distribute to TRPM5 cell microdomains that are not discernable without ultrastructural analysis. In addition to differences in subcellular localization, voltage-gated sodium channels exhibit distinct activation and inactivation profiles. SCN2A and SCN3A inactivate rapidly and contribute to repetitive action potentials, whereas SCN9A persistent currents, stemming from inherent slow closed-state inactivation, augment depolarizing stimuli [34,54]. Thus, expression of multiple voltage-gated sodium channels in TRPM5 cells would allow for precise control of action potential kinetics and waveform.

In addition to voltage-gated sodium channels, taste receptor cells express voltage-gated potassium channels and voltage-gated calcium channels important for firing action potentials [18-20,24,55], as well as voltage-gated chloride channels that may modulate resting membrane potentials and neurotransmission [56]. The molecular identity of these channels is beginning to be elucidated. The voltage-dependent potassium channel KCNQ1 (Kv7.1) was identified in many taste cells, including TRPM5 cells and PKD2L1 cells, whereas KCNH2 (Kv11.1) was identified in a subset of TRPM5 cells [57,58]. The voltage-dependent calcium channel CACNA1A (Cav2.1; α1A) was expressed in PKD2L1 cells, marked with SNAP-25 [59]. The voltage-gated chloride channel CLC-4 was expressed in TRPM5 and PKD2L1 cells, marked with the type 3 inositol 1, 4, 5-triphosphate receptor and SNAP-25 respectively [56]. These numerous voltage-gated ion channels likely contribute to the diversity of action potentials observed in taste cells. TRPM5 cells lack voltage-gated calcium channels [18,19]; thus, action potentials in this cell population consist entirely of depolarizing sodium and hyperpolarizing potassium currents. By contrast, PKD2L1 cells contain voltage-gated sodium, potassium, and calcium channels and action potentials in this cell population consist of sodium and calcium depolarizing currents coupled with potassium hyperpolarizing currents. Depolarization via voltage-gated sodium channels may be required to activate voltage-gated calcium channels. The role of voltage-gated ion channels in amiloride-sensitive and amiloride-insensitive salty taste cell signal transduction is an unexplored area and requires future investigation. Analysis of knockout mice specifically lacking voltage-gated ion channels in taste cells will elucidate the role(s) of action potentials in taste cell function.

Integrating our findings into a current model for taste cell signalling, we propose that activation of SCN2A, SCN3A, and SCN9A in TRPM5 cells generates action potentials and the ensuing depolarization associated with sodium influx stimulates release of ATP neurotransmitter from pannexin/connexin hemichannels to afferent gustatory nerve fibers [38,60,61]. In PKD2L1 cells, activation of SCN2A would generate action potentials and calcium influx via voltage-gated calcium channels would release neurotransmitters by conventional calcium-dependent exocytosis at synapses [36,39].

## Conclusion

SCN2A, SCN3A, and SCN9A voltage-gated sodium channels are expressed in TRPM5 and PKD2L1 taste cells. These voltage-gated sodium channels are positioned to sense the initial depolarizing signals stemming from taste receptor activation at the taste pore and convert signals into action potentials coding taste information to the nervous system.

## Methods

### Laser Capture Microdissection (LCM)

FG and CV taste tissue from 6–8 week old male C57BL/6 mice (Harlan) was embedded in cryomolds using OCT freezing medium (Triangle Biomedical Science) and frozen in an isopentane-dry ice bath. Tissue sections (10–12 um thick) were cut on a Leica CM1850 cryostat, collected on RNase-free membrane slides (Molecular Machines and Industries, MMI), and stained with cresyl violet using the Ambion LCM staining kit as per the manufacturer's instructions. Taste bud and lingual epithelial areas were isolated using a MMI Cellcut laser microdissection system on an Olympus IX71 inverted microscope and collected on MMI reaction tube adhesive lids. Following collection, total RNA from taste bud and lingual areas was separately purified using a Qiagen microRNeasy kit and evaluated using an Agilent 2100 Bioanalyzer with a Series II RNA 6000 Pico Assay. We collected taste bud sections and surrounding lingual epithelial cell areas, pooled from four different mice, for molecular analysis of taste gene expression.

### RNA Amplification and RT-PCR

Total RNA from taste bud and lingual epithelial samples was linearly amplified using two sequential rounds with a RiboAmp RNA Amplification Kit (Molecular Devices) as per the manufacturer's instructions. PCR reactions were set-up in Platinum PCR SuperMix 96 well Non-Skirted PCR plates (Invitrogen) using cDNA synthesized from amplified RNA with random hexamers and the Superscript III First-Strand Synthesis System for RT-PCR (Invitrogen). PCR reactions contained 25 ul of PCR SuperMix, 1 ul of primer pair (see Table 1 for summary of PCR primers), and ~50 ng of appropriate template (taste bud or lingual cDNA). PCR cycling conditions were: 94°C for 2 min; 40 cycles of 94°C for 20 sec, 55°C for 20 sec, and 72°C for 40 sec; 72°C for 2 min; 4°C. All PCR products were cloned and sequenced to validate that bands corresponded to the expected gene products.

**Table 1:** Nucleotide sequences for primers used in RT-PCR studies.

**Target (Accesion no.)**	**Sequence (5'-3')**	**Amplicon size (bp)**
Gustducin (Gnat3)	AGTACTTCGCAACCACCTCCAT (F)	249
[Genebank: NM_001081143]	GTCACTGCATCAAACACAA (R)	

T1R2 (Tas1r2)	TAGGAAAAGACAGGGGGAGTGG (F)	208
[Genebank: NM_031873]	GGGGGTGTAGAGAAGCGAGAAT (R)	

TRPM5	CTCCCAGCAGCCCCAAGAAATG (F)	312
[Genebank: NM_020277]	TGGGTCAGGGGTCAGAAAGAAA (R)	

PKD2L1	GGTGAGATTCCAACAGAGG (F)	202
[Genebank: NM_181422]	CACCACATATTAGTCCAAAAGA (R)	

β-actin	CGTTGACATCCGTAAAGACC (F)	244
[Genebank: NM_007393]	AGGGGCCGGACTCATCGTA (R)	

GAPDH	AGGGCATCTTGGGCTACACTG (F)	310
[Genebank: NM_008084]	TATTATGGGGGTCTGGGATGGA (R)	

SCN1A	CTTTGCTGAGCTGGGTTTGT (F)	227
[Genebank: NM_018733]	GCAGGAGGAAGCGGGGATTTA (R)	
	GTGACTGTGTAAAGGGGAGATA (F)	331
	CACACCGGGAAAAGAGTT (R)	

SCN2A	ATGCGGGAAAATGCTATGAC (F)	325
[Genebank: NM_001099298]	TAACTTTCCACTACTCTACC (R)	
	CAAGAGATTTCAAGGGGAGCAG (F)	298
	TTGGCAAGATCATTTCACTAAC (R)	

SCN3A	AAAGCGGGTCCTGGGTGAG (F)	336
[Genebank: NM_018732]	GGAAGGAGGAGAGGTGGTAGAG (R)	
	ACGCCACCCAGTTCATAGAGTT (F)	290
	CTTGTTTGCGCTTCAGAGTGGT (R)	

SCN4A	GTGGGGCTCTACAGGGCTTGAC (F)	271
[Genebank: NM_133199]	CATGGGGGTGAGAGGAGTAG (R)	
	TTGGGGAACGGGGCTTTGA (F)	223
	GCTGGGGGACCTGGGCTCTTA (R)	

SCN5A	TGTCCCTCCCTGGCACTCACTT (F)	278
[Genebank: NM_021544]	TGCCCCTCCCTCCTTCCGTCTA (R)	

SCN7A	CCATGGCTGCGGGAGAC (F)	239
[Genebank: NM_009135]	CATTTTGCCTTAAGCGGTAGC (R)	
	CCTTCTGTTGGGATTTCTTATT (F)	325
	ATGTCGAGGCAGTGGATTC (R)	

SCN8A	GCAGATGGAGGAGCGGTTCGTG (F)	279
[Genebank: NM_001077499]	CCGCTGCTGCTTCTCCTTGTCG (R)	
	TGGGAGACAGTGGGGAGTTGG (F)	295

	GTCGGGCTTTGTCACGCTGTCG (R)	
SCN9A	AGCCCATACTAGCAGATTCCTC (F)	291
[Genebank: NM_018852]	TATATTCATGGCTACTTACTCA (R)	

SCN10A	AGAGATGGCACCAACCTACAG (F)	302
[Genebank: NM_009134]	ACCCCTATGCGACAGTGC (R)	
	CGTTGGTCCCCGGAGATAAGAT (F)	345
	GGGAGCCCACCGTTGTCATTTG (R)	

SCN11A	GCGAACAAGCGGCGGACTC (F)	287
[Genebank: NM_011887]	TACTTATGGCAGGGTTTTGACT (R)	
	GGAGGAGGAATGTGCCGCTGTC (F)	237
	GTGAGTCCGCCGCTTGTTCGC (R)	

Primers correspond to mouse sequences. F is forward primer and R is reverse primer.

### In Situ Hybridization

Fresh frozen sections (10–12 um thick) were attached to RNase-free SuperFrost Plus slides (Fisher) and processed for in situ hybridization as previously described [62]. DIG-labelled riboprobes were generated for murine TRPM5 (NM_020277; nt 2293–3447), PKD2L1 (NM_181422; nt 838–2417), SCN3A (NM_018732; nt 4414–6028), and SCN2A (NM_001099298; nt 4168–6007).

### Immunohistochemistry

Fresh frozen sections (10–12 um thick), dried at room temperature for 2 hr, were fixed with 4% paraformaldehyde in phosphate buffered saline (PBS) for 20 min. Sections were blocked for 1 hr with 10% normal donkey serum (Jackson Immunoresearch) and 0.3% Triton X-100 for single labelling studies or in 1% tyramide-signal amplification (TSA) blocking reagent (Invitrogen) and 0.1% Triton X-100 for double labelling studies. All antibody incubations were performed in blocking solution. For single label IHC or double label IHC using rabbit and mouse primary antibodies, sections were incubated with both primary antibodies overnight at 4°C, washed with PBS, and incubated with Cy3-conjugated donkey anti-rabbit secondary antibody (single label) and FITC-conjugated donkey anti-mouse secondary antibody (double label) (Jackson Immunoresearch) for 1 hr. Samples were washed with PBS and coverslipped with ProLong Gold antifade reagent (Molecular Probes).

The TSA system was employed to sequentially detect different antigens using two rabbit antisera [63,64]. In this approach, the first primary antibody is diluted below the detection limit of standard IHC methods and detected with a horseradish peroxidase (HRP) labelled secondary antibody that catalyzes the deposition of a fluorescent reaction product. The second primary antibody is then applied at normal levels and detected with a conventional fluorescent secondary antibody. In this manner, the first primary antibody is only detected with the HRP-conjugated secondary antibody. The first antibody was either anti-TRPM5 or anti-PKD2L1 while the second antibody was either an anti-SCN or anti-HCN4. Sections were incubated with TRPM5 or PKD2L1 primary antibodies (using dilutions pre-determined to be below the detection limit of the fluorescent secondary antibody) overnight at 4°C, washed with PBS, and incubated with goat anti-rabbit HRP-conjugated secondary antibody (Invitrogen, TSA Kit #12) for 45 min. Next, sections were washed with PBS, incubated with tyramide-Alexa 488 substrate with hydrogen peroxide for 5 min in the dark as per the manufacturer's instructions, washed with PBS again, and incubated with unconjugated donkey anti-rabbit F(ab)_2 _(Jackson Immunoresearch) overnight at 4°C to quench residual primary antibody. Sections were then reblocked with 10% normal donkey serum for 1 hr and incubated with the second primary antibody overnight at 4°C. Finally, sections were washed in PBS, incubated with Cy3-conjugated donkey anti-rabbit secondary antibody for 60 min, and mounted as above.

Control experiments were performed to demonstrate that the first dilute primary antibody was not detected with the Cy3-conjugated secondary antibody. To this end, every IHC experiment included sections processed as above with the omission of the second primary antibody; no Cy3 fluorescence was detected, thereby validating the specificity of observed signals. In addition, double label IHC of PKD2L1 with SCN3A, PKD2L1 with SCN9A, or TRPM5 with HCN4 labelled distinct taste cell populations, further confirming the absence of antibody cross reaction when using two rabbit primary antibodies. For peptide blocking experiments, antibodies (1 μg) were pre-incubated with corresponding immunizing peptides (5–10 μg) for 1 h at 37°C before applying to tissue sections. Control incubations included antibody without peptide and antibody incubated with a non-immunizing peptide as above.

The following antibodies were used: TRPM5 (a rabbit polyclonal antibody was generated against the 167 amino acid C-terminus of human TRPM5 fused to GST; this affinity purified antibody recognizes TRPM5 in immunoblotting and immunocytochemistry applications; see Additional file 1), PKD2L1 (rabbit polyclonal antibody against a cytoplasmic N-terminal peptide of human PKD2L1; Cat # AB9084, Chemicon; this antibody recognizes human and mouse PKD2L1 in immunoblotting and immunocytochemistry applications), ZO-1 (a mouse monoclonal antibody against amino acids 334–634 of a human recombinant ZO-1 fusion protein; Cat # 33–9100, Zymed), SCN3A (a rabbit polyclonal antibody against a peptide corresponding to amino acids 511–524 in the cytoplasmic loop between domain I and II of rat SCN3A; Cat # AB5208, Chemicon; this antibody is not known to cross-react with any other SCN channels), SCN9A (a rabbit polyclonal antibody against a peptide corresponding to amino acids 446–460 in the cytoplasmic loop between domain I and II of rat SCN9A; Cat # AB5390, Chemicon; this antibody is not known to cross-react with any other SCN channels), SCN2A (a rabbit polyclonal antibody against a peptide corresponding to amino acids 467–485 in the cytoplasmic loop between domain I and II of rat SCN2A; Cat # AB5206, Chemicon; this antibody may cross-react with SCN3A since 12 of 15 amino acids are identical between a region of the SCN2A immunogen and the homologous region of SCN3A), and HCN4 (a rabbit polyclonal antibody against a GST-peptide corresponding to amino acids 119–155 in the cytoplasmic N-terminus of human HCN4; Cat # APC-052, Alomone Labs; this antibody recognizes human and mouse HCN4). SCN antibodies detect known splice variants described in Genbank.

Expression of ion channels in taste bud cells was quantitated by counting the number of immunoreactive taste cells with nuclear profiles in non-adjacent sections from the CV papilla of two to three mice as previously described [65]. Over 200 taste cells from over 30 taste buds were counted per ion channel. The number of immunoreactive cells per taste bud section was summarized as mean +/- SEM in single label IHC studies. In double label IHC experiments, the percentage of labelled cells expressing protein A and protein B, protein A only, or protein B only was determined.

Specimens were viewed on an Eclipse E600 upright microscope (Nikon Instruments, Inc., Melville, New York) equipped with a Plan Fluor 20× objective and a 100 W mercury arc lamp. Fluorescence associated with FITC/Alexa 488 was collected using the following filter sets: 465–495 nm excitation, 505 dichroic beamsplitter, and 515–555 emission; fluorescence associated with Cy3 was collected using the following filter sets: 528–553 excitation, 565 dichroic beamsplitter, and 600–660 emission. Images were acquired using the Spot RT System and Spot v4.6.4.6 software (Diagnostic Instruments, Inc., Sterling Heights, MI), saved in TIFF format, and processed using Adobe Photoshop v9.0.

## Abbreviations

CV: circumvallate; FG: fungiform; HRP: horse radish peroxidase; IHC: immunohistochemistry; ISH: in situ hybridization; LCM: laser capture microdissection; PBS: phosphate buffered saline.

## Competing interests

The authors receive salaries, hold stocks, and are inventors on patents from Senomyx, Inc. and therefore declare competing financial interests.

## Authors' contributions

NG, FE, ML, BL, DK, MEW, PH, AZ and BDM participated in the study design and evaluated data. NG, ML, BL, and DK collected taste cells by LCM. FE isolated RNA and performed RT-PCR experiments. PH and BDM analyzed microarray data. ML and DK generated ISH probes. NG performed ISH and IHC. NG and BDM quantitated IHC data. MEW designed and validated the TRPM5 antibody. BDM drafted the manuscript. All authors read and approved the manuscript.

## Supplementary Material

Additional file 1 **TRPM5 antibody detects TRPM5 in transfected HEK293 cells.** (A) TRPM5 antibody detects TRPM5 protein by Western blotting in transiently transfected HEK293 cells. No signal is observed in mock transfected cells (in a non-adjacent lane on the same gel). β-actin serves as a loading control and shows similar total protein levels in the two samples. Monomeric and dimeric forms of TRPM5 are indicated. (B) TRPM5 antibody stains TRPM5 by immunofluorescence microscopy in stably transfected HEK293 cells. Top panels show brightfield images and bottom panels show fluorescent images. Inset shows magnification of TRPM5 immunoreactive cells. No staining is observed in parental HEK293 cells. Scale bar is 50 μm for panels and 15 μm for inset. Click here for file
