# Supplementary figures and images for: Voltage-gated sodium channels in taste bud cells
Source: BMC Neurosci. 2009 Mar 12;10:20. doi: 10.1186/1471-2202-10-20 (PMC2660338; doi:10.1186/1471-2202-10-20)

# Additional file 1

**A**

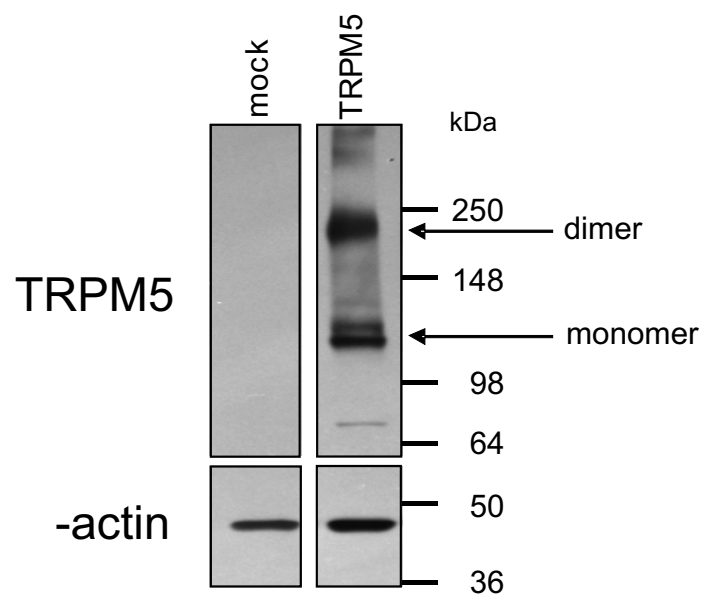

**B**

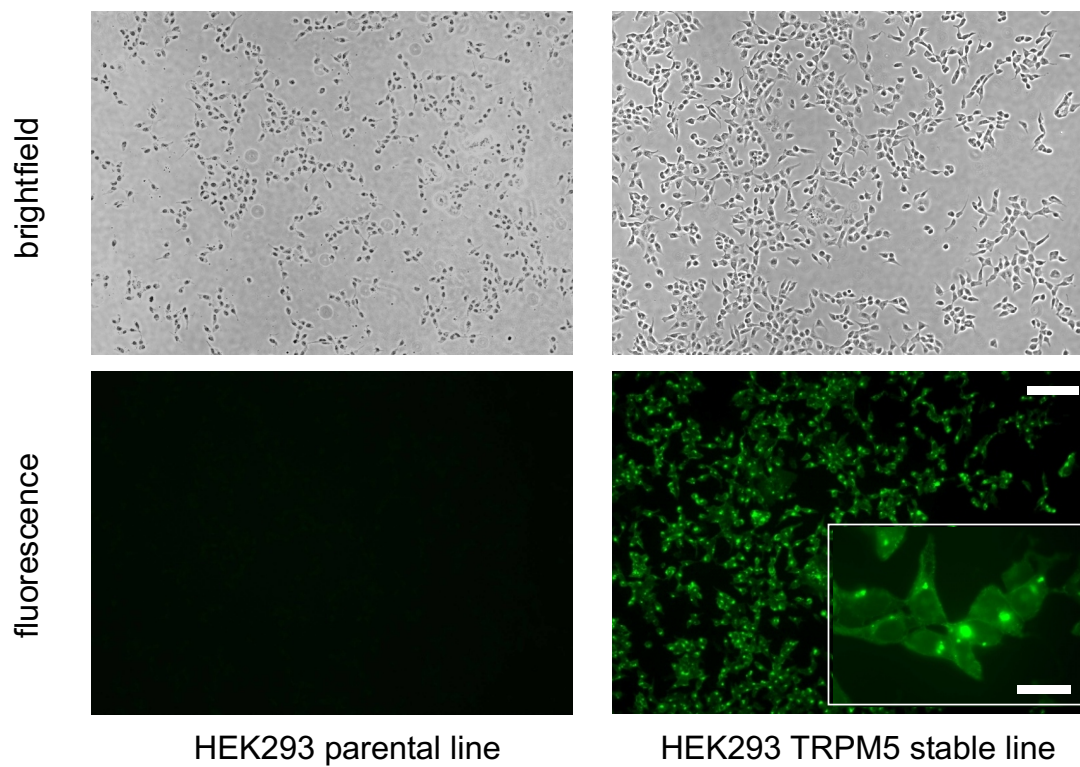

Supplement: Additional file 1 — TRPM5 antibody detects TRPM5 in transfected HEK293 cells. (A) TRPM5 antibody detects TRPM5 protein by Western blotting in transiently transfected HEK293 cells. No signal is observed in mock transfected cells (in a non-adjacent lane on the same gel). β-actin serves as a loading control and shows similar total protein levels in the two samples. Monomeric and dimeric forms of TRPM5 are indicated. (B) TRPM5 antibody stains TRPM5 by immunofluorescence microscopy in stably transfected HEK293 cells. Top panels show brightfield images and bottom panels show fluorescent images. Inset shows magnification of TRPM5 immunoreactive cells. No staining is observed in parental HEK293 cells. Scale bar is 50 μm for panels and 15 μm for inset. [file 1471-2202-10-20-S1.pdf]
